# Floral Initiation in Response to Planting Date Reveals the Key Role of Floral Meristem Differentiation Prior to Budding in Canola (*Brassica napus* L.)

**DOI:** 10.3389/fpls.2016.01369

**Published:** 2016-09-14

**Authors:** Yaofeng Zhang, Dongqing Zhang, Huasheng Yu, Baogang Lin, Ying Fu, Shuijin Hua

**Affiliations:** Institute of Crop and Nuclear Technology Utilization, Zhejiang Academy of Agricultural SciencesHangzhou, China

**Keywords:** canola yield, floral meristem, floral induction, planting date, floral organs

## Abstract

In *Brassica napus*, floral development is a decisive factor in silique formation, and it is influenced by many cultivation practices including planting date. However, the effect of planting date on floral initiation in canola is poorly understood at present. A field experiment was conducted using a split plot design, in which three planting dates (early, 15 September, middle, 1 October, and late, 15 October) served as main plot and five varieties differing in maturity (1358, J22, Zhongshuang 11, Zheshuang 8, and Zheyou 50) employed as subplot. The purpose of this study was to shed light on the process of floral meristem (FM) differentiation, the influence of planting date on growth period (GP) and floral initiation, and silique formation. The main stages of FM developments can be divided into four stages: first, the transition from shoot apical meristem to FM; second, flower initiation; third, gynoecium and androecium differentiation; and fourth, bud formation. Our results showed that all genotypes had increased GPs from sowing to FM differentiation as planting date was delayed while the GPs from FM differentiation to budding varied year by year except the very early variety, 1358. Based on the number of flowers present at the different reproductive stages, the flowers produced from FM differentiation to budding closely approximated the final silique even though the FM differentiated continuously after budding and peaked generally at the middle flowering stage. The ratio of siliques to maximum flower number ranged from 48 to 80%. These results suggest that (1) the period from FM differentiation to budding is vital for effective flower and silique formation although there was no significant correlation between the length of the period and effective flowers and siliques, and (2) the increased number of flowers from budding were generally ineffective. Therefore, maximizing flower numbers prior to budding will improve silique numbers, and reducing FM degeneration should also increase final silique formation. From the results of our study, we offer guidelines for planting canola varieties that differ in maturity in order to maximize effective flower numbers.

## Introduction

Planting date is a simple but essential agronomic practice during crop production. Recommendation of optimal planting date depends on the combination of several factors including plant variety, temperature suitability, and water availability. In modern canola (*Brassica napus* L.) production systems, planting date should be re-considered because of climatic changes, newly bred canola varieties, modern agricultural developments, and human social activities. Considering climatic changes, global warming is the most notable (Manciocco et al., 2014; Yang and Chen, 2014). One of the consequences of global warming is alteration of the growth period (GP) in crops such as rice (Huang et al., 2009). Because the rice-canola rotation is a widely practiced cropping system in China (Li et al., 2012), delaying the harvest time for rice can correspondingly affect the planting date for canola. To the best of our knowledge, newer canola varieties should be adapted to certain ecological areas, which can vary in terms of temperature, amount of sunshine, moisture availability, etc. Hence, the goal in breeding new varieties must generally meet those requirements. Once a new canola line has been developed, evaluation of many traits, including planting date, is necessary to obtain optimal yield and quality before it is released. As for modern agricultural developments and human social activities, important progress in canola production in China has been achieved by the introduction of mechanization for sowing, fertilizing, and harvesting. Because mechanization is labor saving and highly efficient compared to manual practices (Lu, 2009; Hormozi et al., 2012), many agronomic practices are being replaced, such as substitution of transplanting by direct seeding. However, planting dates for canola differ considerably between the two methods because of differences in the seedling GP from sowing to transplanting. Furthermore, the physiological status of the seedlings is also different between the two sowing practices (Boyhan et al., 2009; Mulyati et al., 2009). Thus, it is essential to re-estimate the planting date of canola varieties from the above-analyzed cases.

Normally, either early or late planting dates are adverse for obtaining high yields. Early planting usually results in larger plants. However, there is a trade-off in the consumption of more fertilizer and moisture (Gormus and Yucel, 2002; Sorizano et al., 2004). It has been reported that delayed planting can reduce yield in many crops, including canola (Chen et al., 2005; Sindelar et al., 2010; Tsimba et al., 2013). In our recent study, we found that the reduction of seed yield in canola was largely associated with a decrease in the number of siliques (Hua et al., 2014). The *B. napus* silique is derived from floral meristem (FM) differentiation, and thereafter by sequential flower organ development and normal fertilization (Martínez-Laborda and Vera, 2009; Ferrándiz et al., 2010). In this context, factors that affect FM differentiation should be directly correlated with the final number of siliques. Plant FM differentiation is determined by a population of stem cells within the shoot apical meristem (SAM; Souer et al., 1996). The SAM is involved in organogenesis, and produces aboveground plant tissues such as new leaves and axillary buds (Bowman and Eshed, 2000). Thereafter, the SAM is ultimately responsible for plant architecture. There are two main processes that occur in the SAM: (1) transition from the SAM to the FM, and (2) FM differentiation. These processes have been well characterized in *Arabidopsis* (Irish, 2010). Differentiation of stem cells in the SAM is regulated by many factors including developmental cues, phytohormones, the environment (i.e., temperature and day length), and their interactions (Pickett et al., 1996; Jouannic et al., 2011; Takacs et al., 2012; Uchida et al., 2013). Although several key factors have been identified that function in stem cell transition and differentiation of the SAM and FM, i.e., *LEAFY*, *APETALA1*, *AGAMOUS*, and *WUSCHEL* (Williams et al., 2005; Yang et al., 2013), knowledge on how the plant perceives environmental cues such as temperature and nutrient supply, and responses to crop nursery practices, i.e., planting date and density, are largely unknown.

When modifying planting date, the major issue encountered for canola genotypes is to readjust its physiological status based on changes in climatic factors such as temperature and sunshine hours. For example, the mean temperature in September is much higher than it is in October in China (Hua et al., 2014). High temperatures might tend to modulate physiological metabolism in the plant cell (Suwa et al., 2010; Djanaguiraman et al., 2014). However, will the altered processes that result from different planting dates affect the timing of the transition from SAM to FM and FM differentiation? If so, then will the variation in FM differentiation affect silique formation? Therefore, the profound impacts of changes in planting date on floral initiation should be investigated, because they are closely associated with canola yield. Hence, the goals of the present study were to (1) dissect the morphology of FM during differentiation, (2) compare the timing of the transition of the SAM to FM and FM duration with respect to different planting dates, (3) analyze the variation in the number of flowers at different growth stages with respect to different planting dates, and (4) define the relationship between FM differentiation and effective silique number.

## Materials and Methods

### Site Description and Crop Management

**FIGURE 1:**
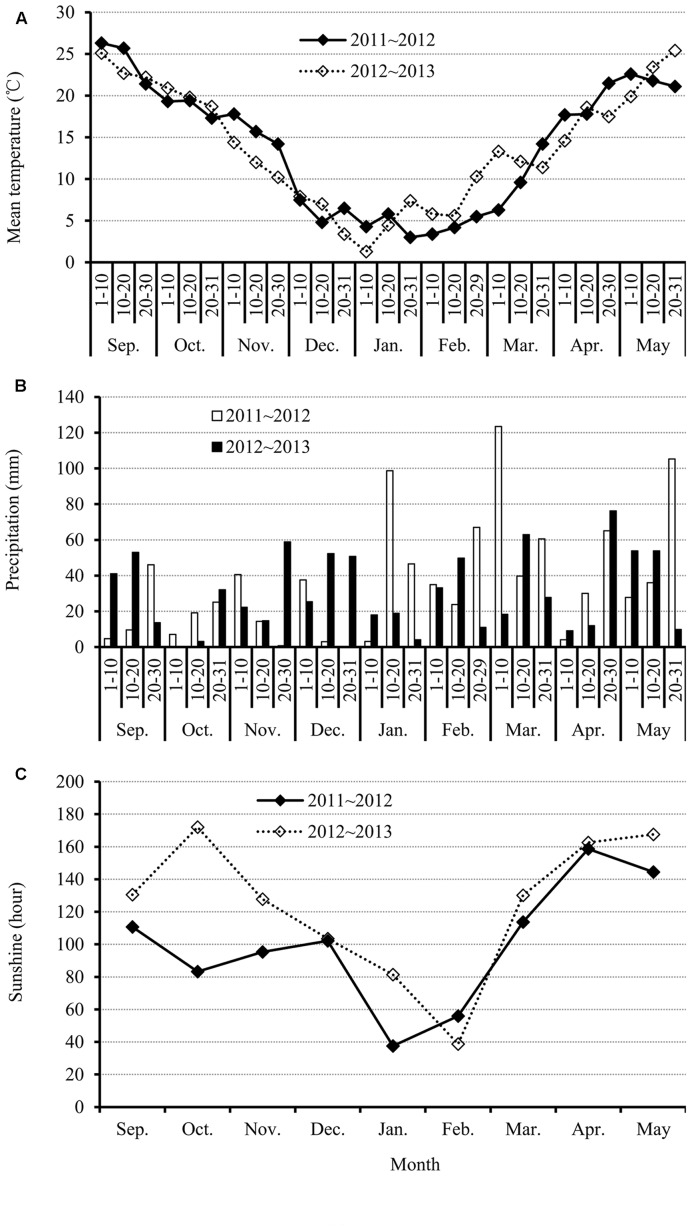
**Dynamics of climatic factors including (A) mean temperature, (B) precipitation, and (C) sunshine hours for each month during the 2011–2012 and 2012–2013 canola growing seasons**.

The experiment was carried out during the 2011–2012 and 2012–2013 growing seasons at the experimental station of the Zhejiang Academy of Agricultural Sciences, Hangzhou, China. Five canola (*B. napus* L.) varieties with different maturities; 1358 (very early), J22 (early), Zhonshuang 11 (middle), Zheshuang 8 (late), and Zheyou 50 (late), were chosen as plant materials. Seeds of 1358, Zhongshuang 11, and Zheshuang 8 were provided by Professor Chunyun Guan, Hunan Agricultural University, while J22 and Zheyou 50 were bred by our research group. The soil type in the experimental station is loamy clay (loamy, mixed, and thermic Aeric Endoaquepts). Canola was rotated with rice and thus the crop previously cultivated was rice. Before sowing, fertilizers including urea, calcium superphosphate, potassium oxide, and borax were broadcast at the rate of 275, 375, 120, and 15 kg ha^-1^, respectively, as a basal fertilizer dose. In addition, urea at the rate of 120 kg ha^-1^ was applied as a topdressing at the end of January in 2012 and 2013. Approximately five canola seeds were directly sown into the soil in each shallow hole in the plot at a depth of approximately 3 cm. After 1 month, the seedlings were thinned to one plant hole^-1^. The field was not irrigated during the canola growing season, and the rainfall profile for the two growing seasons in shown **Figure 1**. Weeds were manually removed at the seedling stage, and aphids were controlled by application of omethoate emulsion 0.06% (V/V) when the plants were finished flowering.

### Experimental Design

The experiment was a split plot arrangement in a randomized complete block design with three replications. Three planting dates; early (15 September), optimal (1 October), and late (15 October), were employed as the main plot while five canola genotypes with different maturities; very early (1358), early (J22), middle (Zhongshuang 11), and late (Zheshuang 8 and Zheyou 50), were surveyed as sub plot. The recommended optimal planting date was considered as the standard with which to compare the effects of different planting dates on agronomic traits and floral initiation. The plants were grown in plots (40 m in length), with spacing between the rows of 0.35 m and spacing between the plants at 0.2 m.

### Imaging the FM Differentiation Process

**FIGURE 2:**
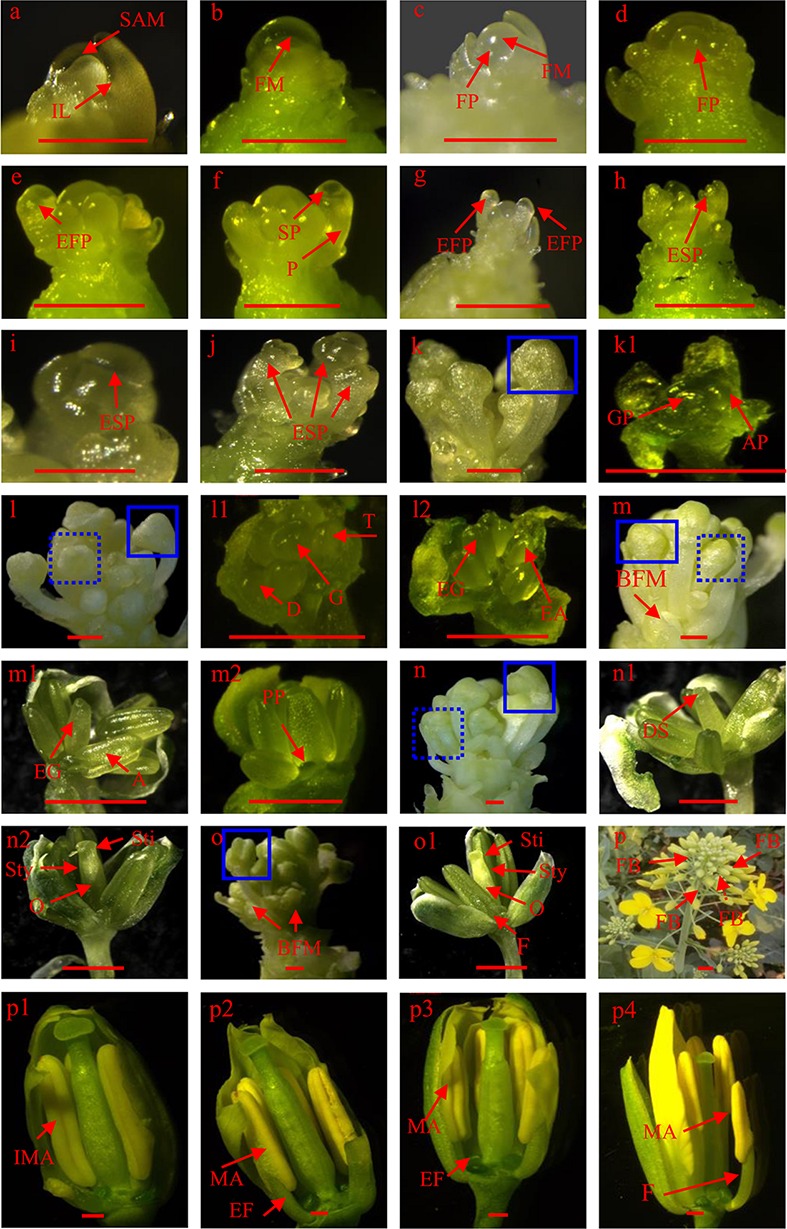
**Floral meristem (FM) differentiation: (a) shoot apical meristem, (b) FM, (c) and (d) floral primordium production on the periphery, (e–j) sepal primordium differentiation, (k–l2) gynoecium and androecium primordium differentiation, (m–o1) petal primordium differentiation and gynoecium and androecium further development, (p) flower bud formation, (p1–p4) anther and stigma maturation**. A, anther; AP, androecium primordium; BFM, branch floral meristem; D, didynamous stamen; DS, dented stigma; EA, elongated androecium; EF, elongated filament; EFP, elongated floral primordium; EG, elongated gynoecium; ESP, elongated sepal primordium; F, filament; FB, flower bud; FM, floral meristem; FP, floral primordium; G, gynoecium; GP, gynoecium primordium; IL, innermost leaf; IMA, immature anther; MA, matured anther; O, ovary; P, pedicel; PP, petal primordium; SAM, shoot apical meristem; SP, sepal primordium; Sti, stigma; Sty, style; T, tetradynamous stamen. Bars equal 1 mm.

To record the FM differentiation process, 10 plants were taken from each plot and delivered to the laboratory for observation of FM differentiation. Roots of the sampled plants were rinsed with water to remove the soil, and all leaves were then removed until they were difficult to distinguish with the naked eye. The removal of the developmental leaves was then performed with a stereomicroscope (Leica M205 C, Germany) using sharp forceps. The growing tip was observed under magnification, and all leaves that enclose the FM were then removed. The morphology of the SAM, FM, and the process of FM differentiation were photographed using software that accompanied the stereomicroscope. The criterion for establishment of a specific differentiation stage (interpreted in the Results section, **Figure 2**) was that >50% of plants enter the given stage.

### Determination of Plant Growth Periods (GPs) for Canola

Data for the canola plant GP was recorded for the different planting dates and included sowing, transition from SAM to FM, budding (BBCH51, Lancashire et al., 1991), initial flowering (BBCH60), middle flowering (BBCH65), end of flowering (BBCH69), maturation (BBCH89), and harvesting (BBCH99). The GP was defined as the interval between two sequential stages. The estimation standard for each growth stage is described as follows: (1) transition from SAM to FM – a first lateral outgrowth was observed on the periphery of meristem; (2) budding – a flower bud >1 cm was observed; (3) initial flowering – opening of the first flower on a plant; (4) middle flowering – approximately 50% percent of the flowers were open on a plant; (5) end of flowering – all the flower buds were open and the plant had finished flowering. At each stage, when 50% of the plants reached the criterion as described above, it was considered to have entered the designated stage.

### Measurement of Flower Numbers

Canola plants were sampled at the budding, initial flowering, middle flowering, end of flowering, and harvest stages. Five plants in each plot were sampled. The flower buds were counted in a combination of those readily visible to the naked eye and those observed through a stereomicroscope and recorded at each sampled stage. When identified through the stereomicroscope, flower primordia were also recorded as flowers (**Figure 2c**). After cessation of flowering, when siliques were formed, all effective and ineffective siliques and unopened flowers were included during counting. For ease of description, we use the terminology ‘flower numbers’ to include siliques, flowers, and unopened flowers. To reduce the large amount of work required, flowers and siliques were counted on the main inflorescence and the 1st, 4th, and 8th branches on the canola plants, and this included branches from different stem positions.

### Statistics

In this experiment, analysis of variance (ANOVA) on flower numbers, silique numbers, and the ratio of siliques to the maximum number of flowers was analyzed using the SAS PROC MIXED procedure (SAS Institute, 2004), where planting dates and canola genotypes were considered to be fixed effects and the year (because the experiments were conducted in the same plots), and replication and all their interactions were considered to be random effects. Further comparisons of multiple means for significance on flower numbers under three planting dates and five genotypes (Supplementary Table S1) at each developmental stage including budding, initial flowering, middle flowering, end of flowering, and maturation were performed using Duncan’s method (*P*-value < 0.05). Furthermore, siliques at harvest stage, and the ratio of siliques to the maximum number of flowers of five genotypes on three planting dates were compared using Duncan’s method (*P*-value < 0.05) as well.

## Results

### Mean Temperature, Precipitation, and Sunshine Hours during the Canola Growing Seasons

Growth of canola requires optimal temperatures, soil moisture (precipitation), and light. However, these climatic factors constantly fluctuate, and their interactions differ from year to year. Thus, the canola plants need to adjust their responses to cope with the environmental changes. Mean temperature varied between the two growth seasons (**Figure 1A**). The apparent differences occurred during four time periods; in November, from 20 December to 10 January, from 20 January to 20 March, and from 20 March to 20 May. The four time periods during 2011–2012 showed higher mean temperature except from 20 January to 20 March. During the temperature recovery stage, which is normally from the end of January, although both years showed increasing trend, the mean temperatures in 2012–2013 were much higher than in 2011–2012. The maximum difference of mean temperature between the 2 years reached 7°C. The fluctuations in temperature during the canola growing season could affect many developmental processes, including floral organ initiation.

For precipitation in the two growth seasons, there were several properties observed in **Figure 1B**. First, the precipitation was distributed very unevenly in different months over the year. For example, the amount of precipitation in January and March was considerably more than in other months in 2011–2012. Second, the differences in precipitation in the same month between the two growing seasons were very large. For example, in certain periods, such as from 20 to 30 November, precipitation in 2012–2013 reached ~60 mm while in 2011–2012 it was zero. However, some periods showed the opposite trend. For instance, a very high level of precipitation was measured (~100 mm) from 10 to 20 January in 2011–2012, but it was only ~20 mm in 2012–2013 (**Figure 1B**).

Sunshine hours exhibited a decreasing trend from autumn to winter, but then increased, from winter to spring, in both growth seasons (**Figure 1C**). However, the number of sunshine hours from September to November in 2011–2012 were much less than in 2012–2013, especially in October, which showed an increase of >80 h of sunshine in 2012–2013 (**Figure 1C**).

The data in **Figure 1** clearly shows that climatic factors, mainly the mean temperature, precipitation, and sunshine hours, were considerably different in the two growing seasons and the canola plants need to adjust their responses to this environmental variation.

### Floral Meristem (FM) Differentiation in Canola

The major steps in canola FM differentiation are illustrated in **Figure 2** which can be divided four main stages. First, SAM transits to FM. Pior to differentiation, the SAM appears conical in shape (**Figure 2a**). Morphological landmarks in the transition from SAM to FM are (1) the meristem becomes much rounder, and (2) bulges form on the periphery of the meristem (**Figures 2b,c**). Second, flower initials from flower primordium. Once FM differentiation has initiated, many outgrowths, namely flower primordia, are produced and surround the meristem (**Figure 2d**). As more new primordia are produced, the outermost flower primordium begins to elongate, and differentiates into a pedicel from the basal part to the bubbled flower primordium (**Figure 2e**). After the pedicel has further elongated, a new protrusion forms in the middle of the bulge, which is the sepal primordium and will develop into a sepal (**Figure 2f**). As elongation of the flower primordium increases (**Figure 2g**), the sepal primordium gradually elongates as well (**Figure 2h**). The four sepals do not develop at the same speed because two of them are obviously longer than the others (**Figure 2i**). Third, gynoecium and androecium differentiate. After the bulge is thoroughly enclosed by sepals (**Figure 2k**), the gynoecium and androecium rapidly undergo differentiation (**Figures 2k,k1**). However, we observed that there are only four androecia. Furthermore, the morphology of the gynoecium and androecium primordia was very similar and was bulged as well (**Figures 2k,k1**). As development of the flower bud progressed, the longer sepals covered the bulged flower primordium (**Figure 2l**), didynamous stamens formed in the younger flowers, and the top of the gynoecium became dented (**Figure 2l1** and the dashed box in **Figure 2l**). In older flowers, both the gynoecium and androecium elongated and a vertical dent appeared in the androecium (**Figure 2l2** and solid box in **Figure 2l**). As the flower bud aged, the shorter sepals became further elongated and branches also began to develop on the FM (**Figure 2m**). At this stage, the gynoecium was slightly taller than the androecium and the top of the gynoecium was still dented in younger flowers (**Figure 2ml** and the dashed box in **Figure 2m**). However, the lengths of the gynoecium and androecium are almost the same in older flowers. Moreover, the petal primordium was easily observed. Until this time, the elongated genoecium was cylindrical (**Figure 2m2** and the solid box in **Figure 2m**). Characteristic of the more developed flower bud was that the four sepals were very apparent and the lengths became similar (**Figure 2n**). The upper part of the gynoecium developed a crack and was much thinner than the lower part in younger buds (**Figure 2n1** and the dashed box in **Figure 2n**). Three parts, the stigma, style, and ovary, were clearly divided into older flowers (**Figure 2n2** and the solid box in **Figure 2n**). Before budding, older flowers become much more mature (**Figure 2o1** and the solid box in **Figure 2o**). Furthermore, the stigma, style, and ovary are more well defined, and the filaments start to differentiate in older flowers (**Figure 2o1**). At this point, all organs in the older buds of the main inflorescence are fully formed and awaiting suitable temperatures to induce budding. Fourth, bud forms. After budding (**Figure 2p**), the main variations in the flower organs are found in the androecium. As the buds age, the anthers are initially yellow–green in color and the filaments are short (**Figure 2p1**). The anthers then turn yellow. Although the filaments become longer, the length of the anther and the filament is still shorter than the stigma at the p2 stage. At the p3 stage, the anther and stigma are almost the same length. Before the flowers open, the mature anther is above the stigma, which ensures pollen grain release and pollination (**Figure 2p4**).

### Influence of Planting Date and Genotype on Growth Periods (GPs) in Canola

The GP represents an important developmental stage for plant organ development, environmental response, and nutrient cycling.

**FIGURE 3:**
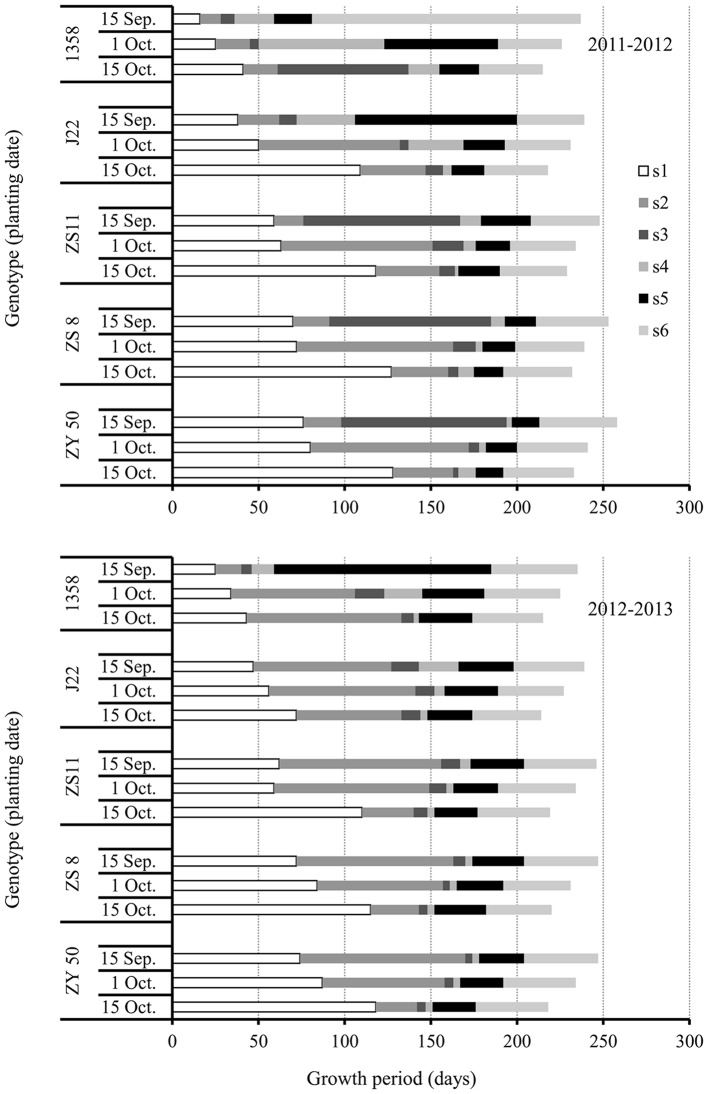
**Growth periods (GPs) during growth of the five canola varieties, 1358, J22, Zhongshuang 11 (ZS11), Zheshuang 8 (ZS8), and Zheyou 50 (ZY50) for three planting dates; early (September 15), optimal (October 1), and late (October 15) in the 2011–2012 (top) and 2012–2013 (bottom) growing seasons. s1, GP from sowing to initiation of FM differentiation; s2, GP from initiation of FM differentiation to budding; s3, GP from budding to initiation of flowering; s4, GP from initiation of flowering to middle flowering; s5, GP from middle flowering to the end of flowering; s6, GP from the end of flowering to harvest**.

Generally, the total growth periods (TGPs) for canola decreased as the planting date were delayed in all genotypes (**Figure 3**). The TGP is composed of six stages; seed sowing to FM differentiation (s1), FM differentiation to budding (s2), budding to initial flowering (s3), initial flowering to middle flowering (s4), middle flowering to the end of flowering (s5), and end of flowering to harvest (s6).

The s1 GP in canola varieties increased with the increase in plant maturity for each respective planting date. This result suggested that early maturing canola varieties, such as 1358, completed the transition process from SAM to FM or from vegetative to reproductive growth much quicker than late maturing varieties, and had no strict requirement for low temperature. As for the impact of planting date on the transition from SAM to FM, our results showed that the s1 GPs increased as planting date was delayed in all varieties. However, the extension of the s1 GPs for the late planting date was especially significant. The result suggested that low temperature under late planting can delay the transition from SAM to FM.

The s2 GPs (from FM to budding) varied greatly among years, genotypes, and planting dates (**Figure 3**). In 2011–2012, varieties showed the longest s2 GPs at recommended plant date except 1358, which had the similar s2 GPs for both the optimal and late planting dates. However, the response of s2 GPs to planting date in all canola varieties was very different in the 2012–2013 growing season. For 1358, the s2 GPs increased significantly as the planting date was delayed. Another early maturing variety, J22, showed the longest s2 GP for the recommended planting date and the shortest s2 for the late planting date. However, in ZS11, ZS8, and ZY50, the lengths of the s2 GPs decreased as the planting dates were delayed in 2012–2013. The s2 GPs for the early planting dates were three–fourfold longer than for the late planting dates in ZS11, ZS8, and ZY50. The result revealed that the period from FM to budding was heavily depending on the climatic factors.

The GPs from s3 to s6, J22, ZS11, ZS8, and ZY50 showed much shorter than that of s1 and s2 at optimal and late planting date in 2011–2012 while they showed the same behavior in all planting dates in 2012–2013. For the early maturing variety 1358, the adjustment of GPs was considerable in various years. For example, the GP from end of flowering to maturation (s6) in the 2011–2012 growth season was very long for this variety, while the longest period was from middle flowering to end of flowering (s5) in the 2012–2013 growth season.

Taken together, although TGPs were similar between the two growing seasons in canola varieties that differed in maturity, the individual GPs were greatly influenced by planting date and year.

### Impact of Planting Date and Genotype on Flower Numbers

Flower numbers were counted from budding to maturation stages in the five canola varieties. Result of ANOVA showed that effect of planting date on flower numbers were not significant but significantly affected by genotype, year and their interactions at budding stage (**Table 1**). At other stages, flower numbers were strongly influenced by planting date and genotype and their interactions (**Table 1**).

**Table 1:** Analysis for variances (planting date, genotype, and year) at different growth stages (budding, BBCH 51; initial flowering, BBCH60; middle flowering, BBCH65; end of flowering, BBCH69; and maturation, BBCH89) (*P* > *F*).

Source	Budding	Initial flowering	Middle flowering	End of flowering	Maturation
Planting date (PD)	0.488	<0.001	<0.001	<0.001	<0.001
Genotype (G)	<0.001	<0.001	<0.001	<0.001	<0.001
Year (Y)	<0.001	<0.001	0.292	<0.001	0.237
PD^∗^G	<0.001	<0.001	<0.001	<0.001	<0.001
PD^∗^Y	<0.001	0.074	0.268	<0.001	0.083
G^∗^Y	0.018	<0.001	<0.001	<0.001	<0.001
PD^∗^G^∗^Y	<0.001	<0.001	<0.001	<0.001	<0.001

**FIGURE 4:**
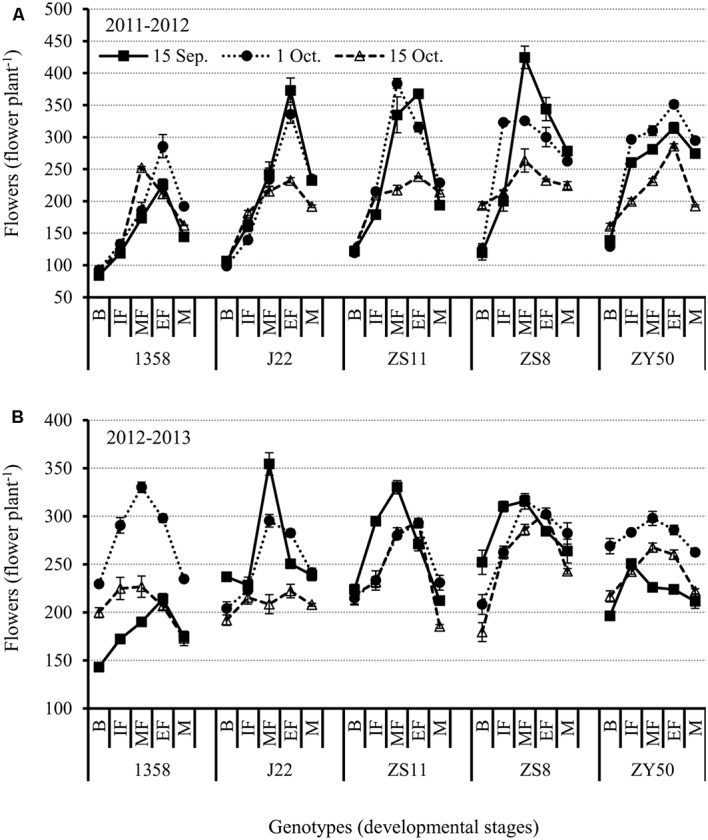
**Changes in flower numbers at different reproductive stages in the five canola varieties 1358, J22, Zhongshuang 11 (ZS11), Zheshuang 8 (ZS8), and Zheyou 50 (ZY50) for three planting dates; early (September 15), optimal (October 1), and late (October 15) in the 2011-2012 (A) and 2012-2013 (B) growing seasons**. B, budding stage; IF, initiation of flowering stage; MF, middle flowering stage; EF, end of flowering stage; and M, maturation stage. Bars indicate standard errors of the mean.

Results showed that flower numbers increased as the canola plants developed, and that flower numbers generally peaked at the end of the flowering stage (**Figures 4A,B**). This showed that FM differentiation is a continuous process that does not end at the budding stage. Considering the effect of planting date on flower numbers, we found the fewest flowers on plants from the late planting date except for the very early variety 1358 (**Figures 4A,B**). In fact, plants of variety 1358 from the early planting date had the fewest flowers. This result suggests that the adverse impact of early planting on very early maturing varieties is much more profound than it is for later planting dates, while the opposite trend was observed for the other four varieties.

In examining the influence of genotype on flower numbers, we found that ZS8 had the maximum number of flowers for the early planting date, while 1358 had the fewest flowers in the 2011–2012 growing season (**Figures 4A,B**). However, the early variety J22 had the maximum number of flowers for the early planting date while ZY50 had the fewest in the 2012–2013 growing season. The relative differences between the 2 years were 100 and 50 flowers, respectively. However, when the flowers were counted at harvest, we found that ZS8 and ZY50 ranked first and second in both years for the recommended planting date (**Figures 4A,B**; Supplementary Table S1).

The results of our study show that the appropriate planting date for very early maturing canola varieties is 1 October because both early and late planting dates can reduce the number of flowers. However, the early-, middle-, and late-flowering canola varieties had the greatest potential to produce more flowers at early planting date.

### Effect of Genotype and Planting Date on Silique Formation

The eventual outcome of flower primordium differentiation is silique formation. Therefore, we counted the total number of siliques on the main inflorescence and the first, fourth, and eighth branches at the harvesting stage.

**FIGURE 5:**
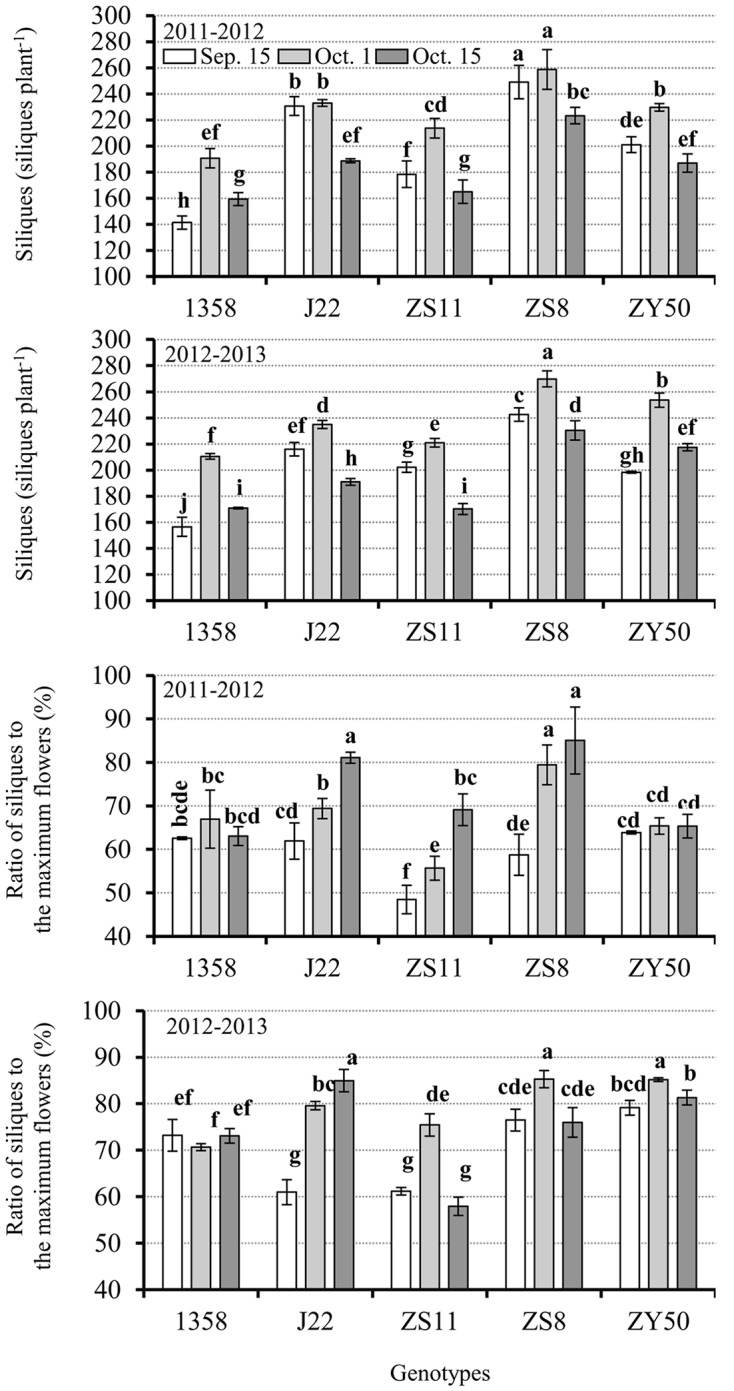
**Silique numbers and the ratios of siliques to the maximum number of flowers at harvest in the five canola varieties 1358, J22, Zhongshuang 11 (ZS11), Zheshuang 8 (ZS8), and Zheyou 50 (ZY50) for three planting dates; early (September 15), optimal (October 1), and late (October 15) in 2011–2012 and 2012–2013.** Different letters on each histogram indicate significant differences at 5% level using Duncan’s test.

In general, more siliques were formed on plants from the recommended planting date than from any other planting date (**Figure 5**). However, the effect of early and late planting dates on silique numbers depended on the canola genotype. For example, the very early variety, 1358, had more siliques from the late planting date, and showed a 10% increase on average for the 2 years as compared with the early planting date. For the other canola varieties, the early planting date was advantageous for silique formation in comparison with the late planting date. Considering genotypic variation, ZS8 had the most siliques, producing 264 siliques plant^-1^ on average over the 2 years, with a total of four inflorescences, for the recommended planting date. The very early maturing variety produced the fewest siliques, averaging 200 siliques over the 2 years for the recommended planting date. Therefore, both planting date and genotype can affect silique production in *B. napus*.

The ratio of siliques to the maximum number of flowers was also recorded. The results showed that regardless of the similarity in the number of siliques formed on plants from different planting dates with different genotypes, the ratio of siliques to the maximum number of flowers varied drastically between the 2 years (**Figure 5**). In general, all canola varieties, except for the very early maturing variety (1358), exhibited higher silique formation ratios for the late planting date in 2011–2012. However, the ratio of siliques to the maximum number of flowers for the late planting date in 2012–2013 was less than for the other two planting date for ZS11, ZS8, and ZY50. The higher silique formation ratio in the 2011–2012 growing season was due to the lower maximum silique numbers. J22 had a higher silique formation ratio for the late planting date, exceeding 80%, for both years. In addition, ZS8 had a higher silique formation ratio at the recommended planting date that averaged >80% in 2012–2013.

## Discussion

Our study focused on the morphology of FM differentiation, the timing and duration of FM differentiation, and floral organ initiation with respect to three different planting dates in canola. Flower and silique formation are the most important issues for canola production because they directly affect seed yield. The adverse influence of delayed planting on canola seed yield has been assessed previously (Holman et al., 2011); however, there are fewer relevant reports describing how floral initiation is affected by different planting dates.

The flower is derived from a flower primordium. Before flower primordium differentiation, the meristem located in the inmost leaves is the SAM, not the FM (Irish, 2010). Therefore, only the SAM transitions into the FM, and a number of flower primordia can be produced and the floral organs such as sepal, pistil, anther, carpel, stigma, and petal will form sequentially. The morphology and process of FM differentiation among the different canola genotypes in this study are the same. Therefore, we only provide an overview of the process of FM differentiation. This suggests that the difference of canola genotypes normally occurs at the amount of flowers but not morphology. Initiation of the transition from SAM to FM is evident from **Figures 2a–c** as described in the section “Results.” In *Arabidopsis*, similar morphological changes, such as an outgrowth produced on the flank of flower primordium, were observed (Smyth et al., 1990). Once the FM differentiation had initiated, the main processes observed were the production of new flower primordia on the periphery of the FM, and the development of the flower primordia into flowers through differentiation of each organ on the outermost flower primordia simultaneously. We observed that these processes in canola were generally similar to those in the related model plant *Arabidopsis thaliana* (Smyth et al., 1990). The other important issue was that of branch inflorescence formation during floral organ initiation in the main inflorescence (**Figures 2m,o**). Although we yet do not know how many branch inflorescences are formed during development of the main inflorescence, we deduced that this aspect of the branch inflorescence is genetically controlled. Furthermore, we also inferred that the branch inflorescences that developed at this stage were effective, because the lower position leaves also contain axillary buds, but they are normally dormant and cannot developed into branches. Branching is also an intricate regulatory network that is affected by several phytohormones such as auxin and cytokinin (Bainbridge et al., 2005; Stirnberg et al., 2012). Therefore, further analysis should focus on the formation of effective inflorescences during development of the main inflorescence to determine whether they are affected by agronomic practices such as planting date, because an increase in the number of effective branch inflorescences can also increase the total numbers of flowers and siliques.

**FIGURE 6:**
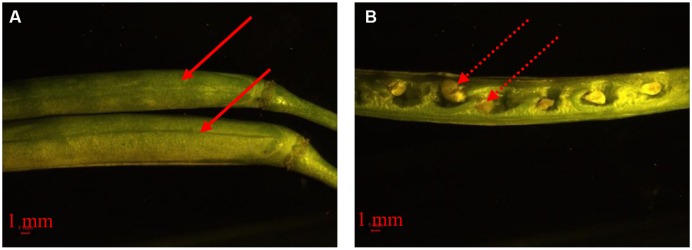
**Morphology of developing siliques (A) and seeds (B) in response to low temperature stress.** The solid arrow means the siliques of the very early maturing variety, 1358, from different branches in early January. The dashed arrow means aborted developing seeds.

The development of floral organs is affected by many environmental and agronomic practices. The planting date triggers the appropriate initiation and timing of FM differentiation. In canola, the transition from SAM to FM is the prelude to the reproductive stage. In our study, the very early variety showed rapid transition from SAM to FM. The quick transition from vegetative to reproductive growth can have severe consequences. First, rapid transition often results in lower accumulation of plant biomass and hence the nutrient supply from leaf and stem compared to the recommended planting date (unpublished date). During floral development, a considerable level of nutrients should be absorbed from other tissues (Sklensky and Davies, 2011). Consequently, flower organ formation may be predictably decreased. A second consequence is low temperature stress on flower and silique development. As seen in **Figures 1** and **3**, the very early maturing variety encountered low temperatures during flowering and silique development. The influence of low temperatures on flowering and silique development can be destructive (Wingler, 2011). We observed that developing seeds aborted after low temperature stress (**Figure 6**). Therefore, early planting should be avoided for the very early maturing canola varieties. Even though more flowers and siliques formed on plants from the late planting date than the early planting date for the very early maturing variety, late planting is not recommended. When the very early maturing variety was planted late, the timing from vegetative to reproductive stage increased because of decreasing temperatures in November. It has been reported that low temperature can retard or even terminate FM differentiation (Sun B. et al., 2009; Liang et al., 2012). However, although the low temperatures slowed the growth of the plants, the very early maturing variety is able to accelerate the initiation of FM differentiation when canola has been exposed to low temperatures to pass vernalization (Adhikari et al., 2012; Wollenberg and Amasino, 2012). Once a suitable temperature is reached, the canola plant can promptly differentiate many flowers with a small vegetative biomass as well. Unlike early planting, the vegetative growth is also vigorous for the very early maturing variety prior to the arrival of low temperatures; however, the very early variety may not develop much biomass, with fewer leaves and branches, which could then lead to reduced flower numbers as compared with plants grown from the recommended planting date.

For the other four canola varieties in this study, the timing of the transition from vegetative to reproductive growth in response to the late planting date was also longer than for either the early or recommended planting dates. At the early and recommended planting dates, the canola varieties required a period of low temperature for vernalization (Filek and Dubert, 1994; Sheldon et al., 2009). Thus, a low temperature stimulus for the transition from vegetative to reproductive growth was very important for all of the canola varieties except for the very early maturing variety 1358. Before low temperature induction, the plants will accumulate vegetative biomass. The moderate level of nutrients stored in leaves and un-elongated stems is a benefit for the early stage development of reproductive organs. However, the early planting should be advanced in some limitation. The most important reason is because late maturing canola varieties cannot go through FM differentiation without low temperature induction. However, for the late planting date, a situation similar to that with the very early maturing variety occurred. But the effect of late planting on FM differentiation in canola was more serious than was early planting for the middle and late maturing varieties. For these varieties, low temperature ended during flowering and silique development (**Figures 1** and **3**). Therefore, it is suggested that planting somewhat earlier for middle and late maturing canola varieties is a feasible practice.

In the current study, there were other important considerations as well. First, budding is a key point both for effective flower production and silique formation on all planting dates. FM differentiation is a continuous process once started (**Figure 4**). All the genotype and planting date treatments showed a similar trend in that the number of flowers increased from budding. However, we also found that >20% of the flowers were ineffective based on the ratio of silique formation. Those ineffective flowers or flower primordia represent a considerable waste of resources. Because flowers and flower primordia are not green tissues, they cannot synthesize carbohydrates via photosynthesis. Before the ineffective flower primordia die, they participate in nutrient recycling in different organs such as the leaf and stem (Guiboileu et al., 2010). Their nutrient uptake could possibly affect the effective flower supply during development. Although silique formation is influenced not only by the flower primordium but also by processes such as flower fertilization, the first step is to produce enough flowers. The relatively low ratio of silique formation might be a feedback mechanism for both the degeneration of the FM and the un-fertilized flowers as the temperatures continue to increase. From this perspective, improving the percentage of effective flowers is very meaningful with respect to silique formation, and thus seed yield. In addition to canola, many plants show similar behavior for flower degeneration and drop (Kaska, 1989; Wang et al., 2012). For example, Alburquerque and Egea (2004) showed that low flower bud production and high flower bud drop often resulted in poor yields in apricot. Although the exact regulatory mechanism underlying flower degeneration and drop is not well understood, some hypotheses had been proposed, such as fertilization, nutrient supply, and phytohormone modulation (Buban and Faust, 1982; Pozo, 2001; Sun Y. et al., 2009; Monerri et al., 2011; Boldingh et al., 2016). Recently, Gamer and Lovatt (2016) reported that on average more 70% flowers in avocado trees cannot be fertilized due to the pollen grains unable to germinate and produce pollen tube resulting in ovule degeneration and flower drop. During this process, ABA concentrations in abscising organs were much higher throughout the early to late drop while IAA and isopentenyladenine was much higher at middle drop stage. The result suggested that different phytohormones may function in distinct way on the regulation of flower drop. Another piece of evidence from Boldingh et al. (2016) showed that flowers with successful fruit set had higher carbohydrates and boron content revealing the importance of these matters for flower development to reduce the drop ratio of flowers. In addition to the ABA, jasmonic acid-like phytohormone was also identified as the similar role on flower and fruit early abscission (Pozo, 2001). Another case, developing flower degeneration occurs because of the competition between different positions of flower. Like other crop such as rice, the flower or seed development is not synchronous. Earlier flower or seed developed usually has stronger opportunity to obtain assimilates compared with younger developing flowers (Fu et al., 2013; Zhang et al., 2014). Furthermore, as temperature increased very quickly after canola flowering (**Figure 1a**), high temperature would together speed up the degeneration of developing flowers (Selak et al., 2014). Therefore, to understand canola flower bud degeneration and flower drop, additional investigations need to be conducted toward improving the effective flower ratio in canola.

**FIGURE 7:**
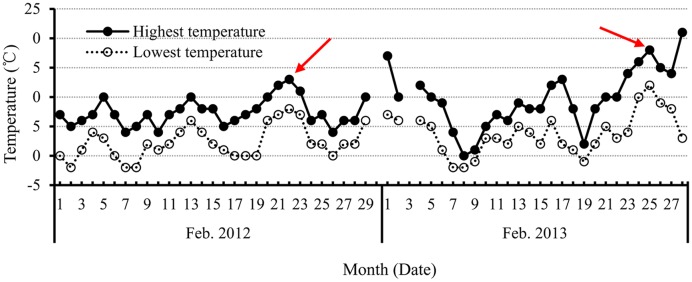
**The highest and lowest temperature each day in February in 2012 and 2013, during the period of FM differentiation in canola.** The red arrow indicates the unusually high temperatures that occurred during the initiation of FM differentiation to budding stage.

The second consideration is how flower numbers, including flower primodia, are associated with the duration of FM differentiation before budding. As discussed above, FM differentiation occurred continuously after budding, but the differentiated flowers were ineffective. In this context, the production of flowers during the stage from FM initiation to budding plays a key role in the total effective numbers of flowers or siliques. Therefore, is there any relationship between flower numbers and the length of the duration from initiation of FM differentiation to budding? Our simple correlation analysis between genotype and flower numbers at the budding stage revealed that only the very early maturing variety showed a significant relationship (*r* = 0.88^∗^). In addition, correlation analysis of planting date and flower numbers at the budding stage showed there was a correlation with early planting date (*r* = 0.92^∗^). The non-significant correlation between flower numbers at budding stage and the length of duration from initiation of FM differentiation to budding revealed it should be a complex process because of the complex climatic conditions such as low temperature, tentative high temperature (**Figure 7**), and others. Therefore, optimizing the developmental conditions, including plant resistance to environmental stress and other strategies to prevent or avoid the effects of adverse climatic changes is a vital way to improve canola flower numbers and, hence, siliques.

**FIGURE 8:**
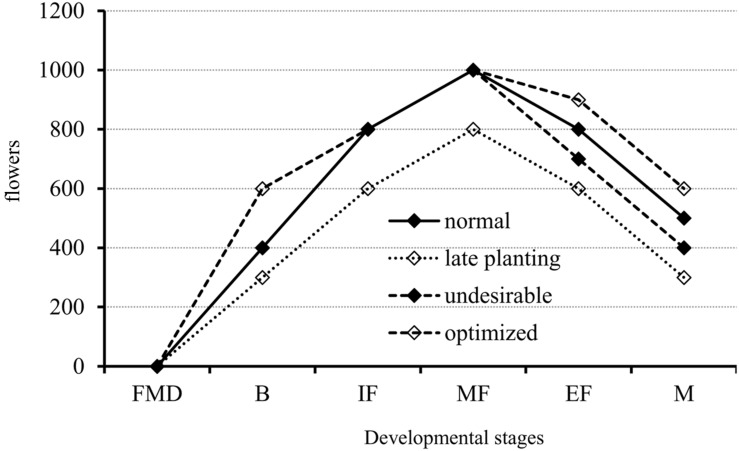
**A proposed model for flower development in canola from the initiation of FM differentiation to maturation in response to normal and late planting dates, and other possibly undesirable conditions such as low temperature stress.** The flower number was not the real one. The dotted line is for the late planting date that showed fewer flowers at all developmental stages as compared with the normal condition (solid line). The dashed line with open diamonds indicates the potential improvement to maximize flower numbers at the budding, end of flowering, and maturation stages. The dashed line with solid diamonds indicates the potential for flower loss when an undesirable environmental condition occurs. B, budding stage; FMD, floral meristem differentiation stage; IF, initiation of flowering stage; MF, middle flowering stage; EF, end of flowering stage; and M, maturation stage.

Therefore, we derived a model for the initiation of canola floral organs from experiments using varieties that differ with respect to maturity and three planting dates (**Figure 8**). As illustrated in **Figure 8**, there are three important stages: budding, middle flowering, and maturation. The first stage, from FM differentiation to budding, is a fundamental period for flower production. During this time, plants of the middle- and late-maturing varieties often produce fewer flowers (dotted line with open diamonds) and have the potential to maximize flower numbers (dashed line with open diamonds). The second stage is from budding to the middle of the flowering period, and flower numbers for all genotypes have peaked (sometimes the peak will shift to the end of flowering). Most of the flowers produced or the developing flowers are ineffective, thus, it is presently unknown whether reducing the number of flowers formed during this time will affect final silique formation. The third stage is from the middle of flowering to maturation. During this stage, canola plants suffer from exposure to undesirable conditions, i.e., low temperature stress for the very early variety, and nutrient deprivation and heat stress for all genotypes, which causes them to produce fewer siliques (dashed line with solid diamonds). However, optimizing flower numbers (dashed line with open diamonds) through genetic manipulation, i.e., screening for genotypes with lower ratios of flower degeneration, and agronomic practices such as appropriate application of plant growth regulators, could very well improve yields in canola.

Conclusively, our study showed a clear morphological change of FM developmental process including FM transition from SAM, flower initiation, gynoecium and androecium differentiation, and bud formation in canola. There were no morphological differences between genotypes and planting dates but with significant differences on flower number. Growth days in canola genotypes with different maturity responded to planting date in a delay mode during sowing to enter FM transition while strongly depended on the year and genotype from FM differentiation to bud. Although flower numbers in different genotypes reduced under delayed planting date condition, all the genotypes showed a close number of flowers between budding and harvesting stage. The result suggested an important period from FM differentiation to budding for effective flower production onto fruit set (silique formation). The continuous increasing of flower numbers from budding in five canola genotypes and low ratio of silique numbers to the maximum flowers indicated a great waste of flower investment. Therefore, maximize the flowers before budding and minimize the flower degeneration and drop after budding is a future research and practice direction for canola yield improvement.

## Author Contributions

YZ performed the floral meristem photographing and wrote the manuscript. DZ design the experiment. HY recorded the growth period at different developmental stages. BL and YF obtained the agronomic trait data. SH design the experiment, data analysis, and manuscript revision.

## Conflict of Interest Statement

The authors declare that the research was conducted in the absence of any commercial or financial relationships that could be construed as a potential conflict of interest.
