# Supplementary material for: Floral Initiation in Response to Planting Date Reveals the Key Role of Floral Meristem Differentiation Prior to Budding in Canola (Brassica napus L.)
Source: Front Plant Sci. 2016 Sep 14;7:1369. doi: 10.3389/fpls.2016.01369 (PMC5021690; doi:10.3389/fpls.2016.01369)
Supplement: Supplementary file 1 [file Table_1.DOCX]

Table S1 comparison of mean value for flower number during 2011-2012 and 2012-2013 growth season under three planting dates (PD1, 15 Sep.; PD2, 1 Oct; and PD3, 15 Oct) at five growth stages (B, budding; IF, initiation flowering; MF, middle flowering; EF, end of flowering; M, maturation) using Duncan’s method. Means followed by the different letters differed at α=0.05

| Genotype | Planting date (PD) | 2011-2012 | | | | | 2012-2013 | | | | |
| --- | --- | --- | --- | --- | --- | --- | --- | --- | --- | --- | --- |
|  |  | B | IF | MF | EF | M | B | IF | MF | EF | M |
| 1358 | PD1 | a | b | b | b | c | c | c | c | b | b |
|  | PD2 | a | a | b | a | a | a | a | a | a | a |
|  | PD3 | a | b | a | b | b | b | b | b | b | b |
| ZS11 | PD1 | a | b | b | a | c | a | a | a | b | b |
|  | PD2 | a | a | a | b | a | a | b | b | a | a |
|  | PD3 | a | a | c | c | b | a | b | b | a | c |
| ZS8 | PD1 | b | b | a | a | a | a | a | a | b | a |
|  | PD2 | b | a | b | b | b | b | b | a | a | a |
|  | PD3 | a | b | c | c | c | c | b | b | a | b |
| ZY50 | PD1 | b | b | b | b | b | c | b | c | c | c |
|  | PD2 | c | a | a | a | a | a | a | a | a | a |
|  | PD3 | a | c | c | c | c | b | c | b | b | b |
| J22 | PD1 | a | b | a | a | a | a | a | a | b | a |
|  | PD2 | b | c | a | b | a | b | a | b | a | a |
|  | PD3 | a | a | a | c | b | c | a | c | c | b |

| Planting date (PD) | Genotype | 2011-2012 | | | | | 2012-2013 | | | | |
| --- | --- | --- | --- | --- | --- | --- | --- | --- | --- | --- | --- |
|  |  | B | IF | MF | EF | M | B | IF | MF | EF | M |
| PD1 | 1358 | d | e | e | d | d | e | e | e | e | d |
|  | ZS11 | b | c | b | ab | c | c | b | b | b | c |
|  | ZS8 | b | b | a | b | a | a | a | c | a | a |
|  | ZY50 | a | a | c | c | a | d | c | d | d | c |
|  | J22 | c | d | d | a | b | b | d | a | c | b |
| PD2 | 1358 | c | d | d | d | e | b | a | a | ab | c |
|  | ZS11 | b | c | a | bc | d | c | c | d | bc | c |
|  | ZS8 | ab | a | b | cd | b | c | b | b | a | a |
|  | ZY50 | a | b | b | a | a | a | a | c | cd | b |
|  | J22 | c | d | c | ab | c | c | c | c | d | c |
| PD3 | 1358 | e | d | a | c | d | b | cd | c | e | e |
|  | ZS11 | c | a | b | b | b | a | bc | a | b | d |
|  | ZS8 | a | a | a | b | a | ca | a | a | a | a |
|  | ZY50 | b | b | b | a | c | a | b | b | c | b |
|  | J22 | d | c | b | b | c | bc | d | d | d | c |
